# Primary penile melanoma and genital lichen sclerosus

**DOI:** 10.1002/ski2.274

**Published:** 2023-08-12

**Authors:** Kate Dear, Georgios Kravvas, Sharmaine Sim, Evanthia Mastoraki, Mariel James, Richard Watchorn, Aiman Haider, Peter Ellery, Alex Freeman, Hussain M. Alnajjar, Asif Muneer, Christopher B. Bunker

**Affiliations:** ^1^ Department of Dermatology University College London Hospitals NHS Foundation Trust London UK; ^2^ University College London Medical School London UK; ^3^ Department of Histopathology University College London Hospitals NHS Foundation Trust London UK; ^4^ Department of Urology University College London Hospitals NHS Foundation Trust London UK

## Abstract

**Background:**

There is a well‐established association between squamous cell cancer and genital lichen sclerosus (GLSc). Although there have been several reported cases of vulval melanoma (MM) associated with LSc, particularly in the paediatric population, fewer cases of male genital (M) GLSc and penile (Pe)MM have been published.

**Objectives:**

The aim of this study was to explore further the relationship between PeMM and MGLSc by reviewing all the cases managed by our multidisciplinary service over a finite period.

**Methods:**

All patients known to our tertiary urology and male genital dermatology service with a diagnosis of PeMM and where histology was available for review were identified over an 11‐year period (2011–2022). The histology was reviewed by two independent, mutually ‘blinded’ histopathologists. Photographs and clinical notes, where available, were retrospectively reviewed by two independent dermatologists for signs or symptoms of LSc.

**Results:**

Eleven patients with PeMM were identified for review. Histopathological examination found evidence of LSc in nine patients, and review of clinical photos corroborated the presence of LSc in three. Overall, features of LSc were present in nine out of eleven cases (82%).

**Conclusion:**

The presence of LSc in 9 out of 11 cases of PeMM is suggestive of a causative relationship between LSc and PeMM. This may be due to chronic melanocytic distress created by chronic inflammation secondary to LSc.

## Graphical abstract

We retrospectively identified all patients with a diagnosis of penile malignant melanoma at our tertiary dermatology and urology centre over an 11‐year period (2011–2022) and reviewed the available histology, medical notes, and clinical photographs for evidence of male genital lichen sclerosus. This was found to be present in nine out of eleven (82%) of cases. We hypothesise the aetiology of this rare malignancy in patients with lichen sclerosus, which itself is an under‐reported and under‐diagnosed condition with significant implications for patients.

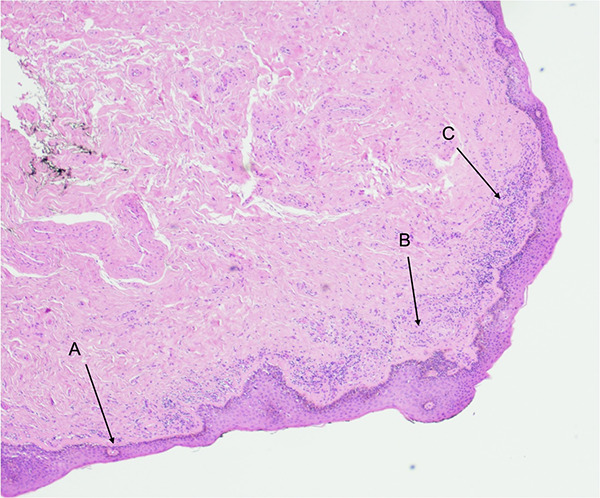

**What is already known about this topic?**

- The association between squamous cell cancer and genital lichen sclerosus (GLSc) is well established. However, despite a number of isolated case reports in the literature, no concrete link was established between lichen sclerosus and genital melanoma.

**What does this study add?**

- This study confirmed the presence of lichen sclerosus in 82% of penile melanomas, suggesting a causal relationship. This association may be due to chronic melanocytic distress created by chronic inflammation secondary to lichen sclerosus. This emerging link has implications for the prevention, early diagnosis, management, and follow‐up of male GLSc.

## 1 INTRODUCTION

The association of squamous carcinoma (SCC) with genital lichen sclerosus (GLSc) is well established.^1^, ^2^, ^3^ However, there is increasing literature concerned with the relationship of GLSc to genital melanoma (MM).^4^ In men this has been confined to case reports.^5^

The aim of this study was to explore further the relationship between penile (Pe)MM and GLSc by reviewing cases of PeMM seen by our multidisciplinary service over a period of 11 years.

## 2 MATERIALS AND METHODS

We retrospectively identified all patients known to our tertiary urology and male genital dermatology service with a diagnosis of PeMM in the period (2011–2022) for whom histopathology was available for review. The relevant patients were searched for based on multidisciplinary team meeting records.

Two dermatologists (Georgios Kravvas, Christopher B. Bunker) reviewed the clinical records and the clinical photographs where available and two histopathologists (Aiman Haider, Peter Ellery) independently reviewed the histology from the material available and recorded features of GLSc where present. The histopathologists were blinded to prior histological reports and clinical records and to each other's reports. They were, however, aware of the topic of the study. In cases of discordance between the two histopathologists, a third independent histopathologist (Alex Freeman) reviewed the cases.

## 3 RESULTS

A total of 11 patients with PeMM were included in our study. Details of these patients are shown in Table 1.

**TABLE 1:** The demographic and clinical findings of the studied patients.

Patient study number	Age at diagnosis (in years)	Ethnicity	Melanoma subtype	Stage	Mutations	Features of LSc on histology	Features of LSc on photos	Features of LSc on medical records	Compelling presence of LSc
1	64	White British	Superficial spreading	pt4b	n/a	Present	n/a	n/a	Present
2	71	White British	Superficial spreading	pt4b	NRAS	Absent	n/a	Phimosis	Absent
3	68	White British	Nodular	pt4b	n/a	Present	Present	Phimosis	Present
4	69	White British	Superficial spreading	pt4b	NRAS	Present	n/a	n/a	Present
5	86	White British	Nodular	pt4b	NRAS	Present	n/a	n/a	Present
6	45	White other	Superficial spreading	pt2b	BRAF	Present	Present	None	Present
7	74	White British	Superficial spreading	pTis	n/a	Present	n/a	None	Present
8	32	Asian Indian	Unknown	n/a	n/a	Present	n/a	Etiolation, tight fraenulum, constrictive posthitis	Present
9	63	White British	Superficial spreading	pt1a	n/a	Present	Present	None	Present
10	80	White British	Superficial spreading	pt2a	n/a	Present	n/a	None	Present
11	79	n/a	Superficial spreading	pt3b	Absent	Absent	n/a	None	Absent

Abbreviation: n/a, not available.

The average age at diagnosis of PeMM was 66 years (range 32–86 years) and the majority of patients (73%) were White British. The most common type of MM seen was superficial spreading MM in eight cases, with nodular MM recorded in two cases. In one case the subtype could not be determined as only tissue from the wide local excision (in which there was no residual MM) was available for histopathological assessment (this patient underwent primary excision at another institution before being referred to our hospital for further management). In five cases (45%), the stage was recorded as pT4b. One case was recorded as pTis, one case as pT1a, one case as pT2a, one case as pT2b and one case as pT3b. BRAF mutation was found in one case and NRAS mutations in three cases. Mutations were not recorded or sought in six cases and were not detected but sought in one case.

Overall, GLSc was present on histopathology in nine out of eleven cases (82%). Histopathology findings for GLSc were concordantly positive in five cases and concordantly negative in 1 case. In the five cases that were disconcordant, a third histopathologist reviewed the cases and deemed GLSc to be present in four cases and absent in one case.

Photographs were available to interpret for three cases. Two clinicians reviewed these photographs, and both found clinical signs in keeping with lichen sclerosus in all three; all three also had histological evidence of LS.

The clinical notes and letters were available for review in eight cases. Symptoms suggestive of GLSc were mentioned in only one case, and signs suggestive of GLSc were documented in three cases. The breakdown of symptoms and signs was as follows: one patient reported a tight prepuce and was found to have phimosis; another one was also found to have phimosis; a third had etiolation of the glans, a tight fraenulum, and constrictive posthitis. In one patient LSc was corroborated histologically, and in one both histologically and on review of the photos. The third patient did not have available photos for review and histologically no LSc was identified. Given the lack of incontrovertible evidence, this patient was not included in the list of those with confirmed LSc.

**FIGURE 1:**
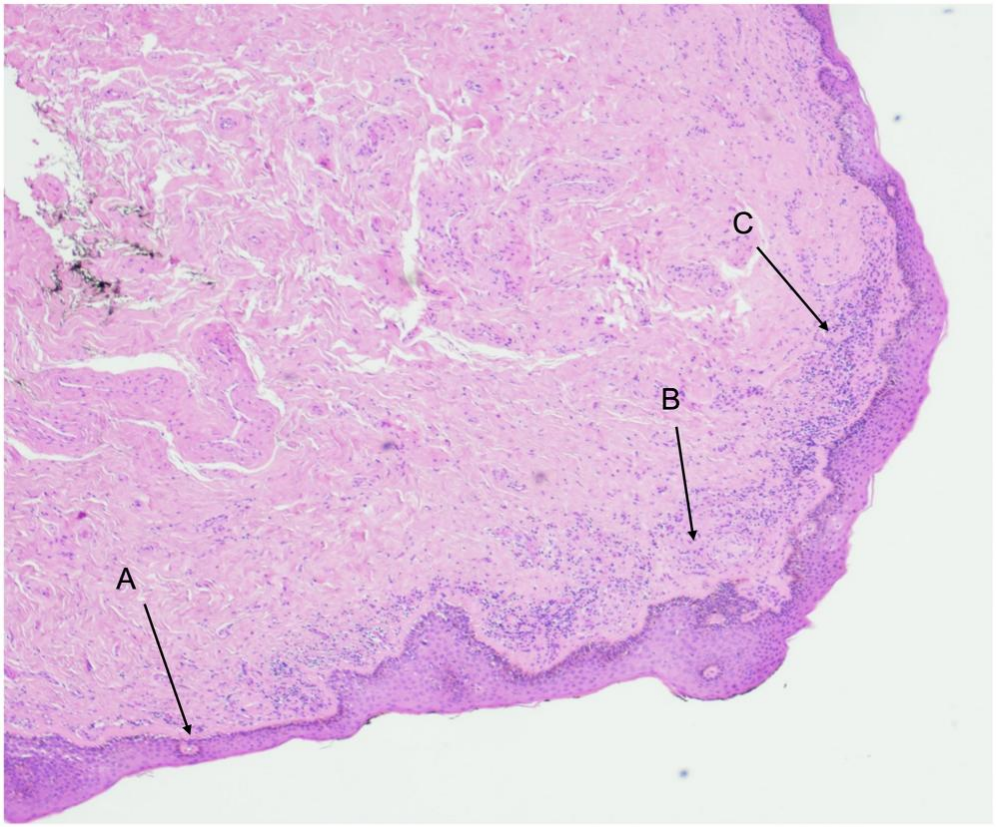
Histological examination of penile tissue. Haematoxylin and eosin staining, original magnification x20. Features in keeping with lichen sclerosus in a 63‐year‐old man with superficial spreading melanoma. (a) Thinning and attenuation of the surface squamous epithelium; (b) Subepithelial sclerosis; (c) Lichenoid chronic inflammatory cell infiltrate.

**FIGURE 2:**
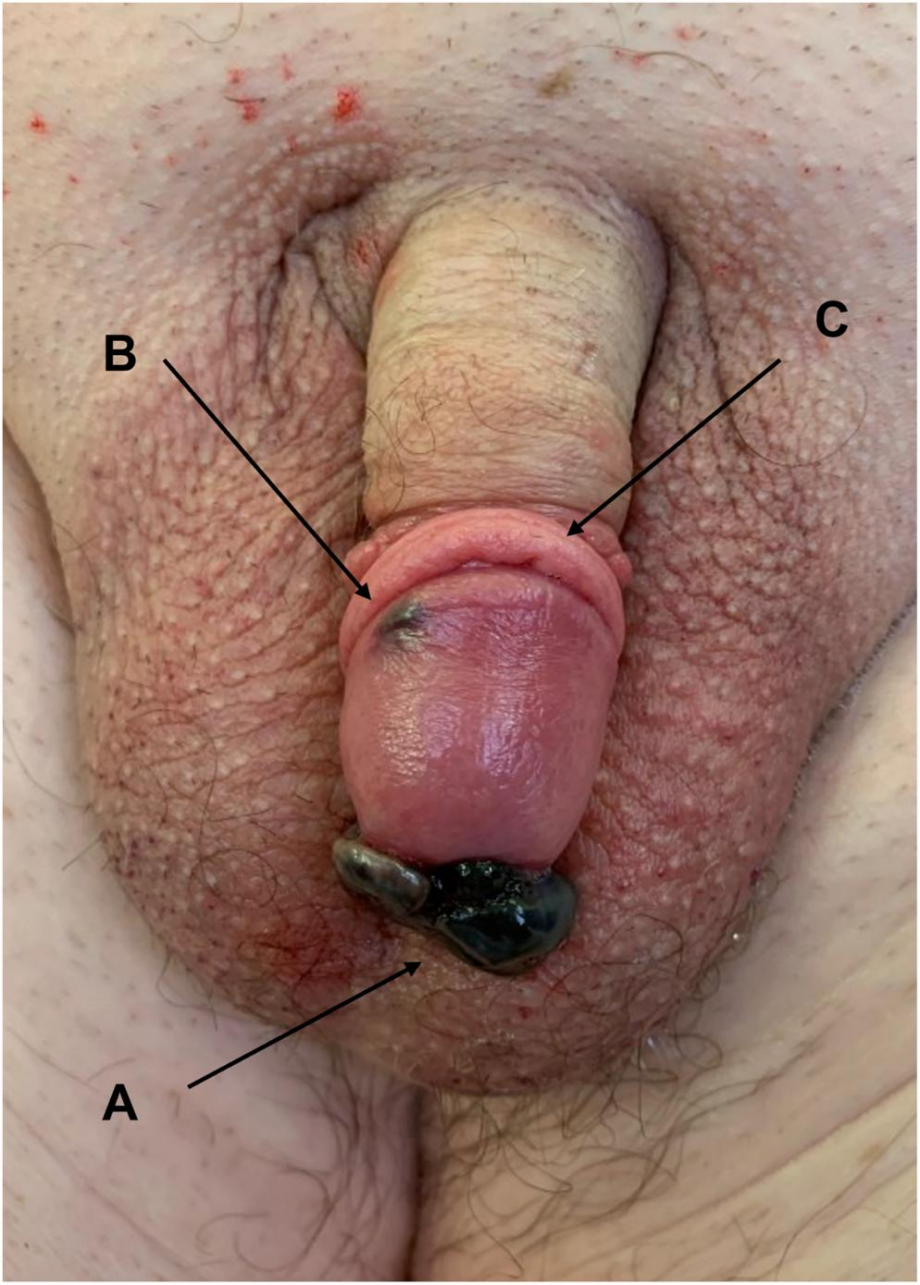
Clinical photograph of a 68‐year‐old man with nodular melanoma of the penis and lichen sclerosus, diagnosed both clinically and histologically. (a) Melanoma; (b) in transit metastasis; (c) Constrictive lichenoid posthitis.

**FIGURE 3:**
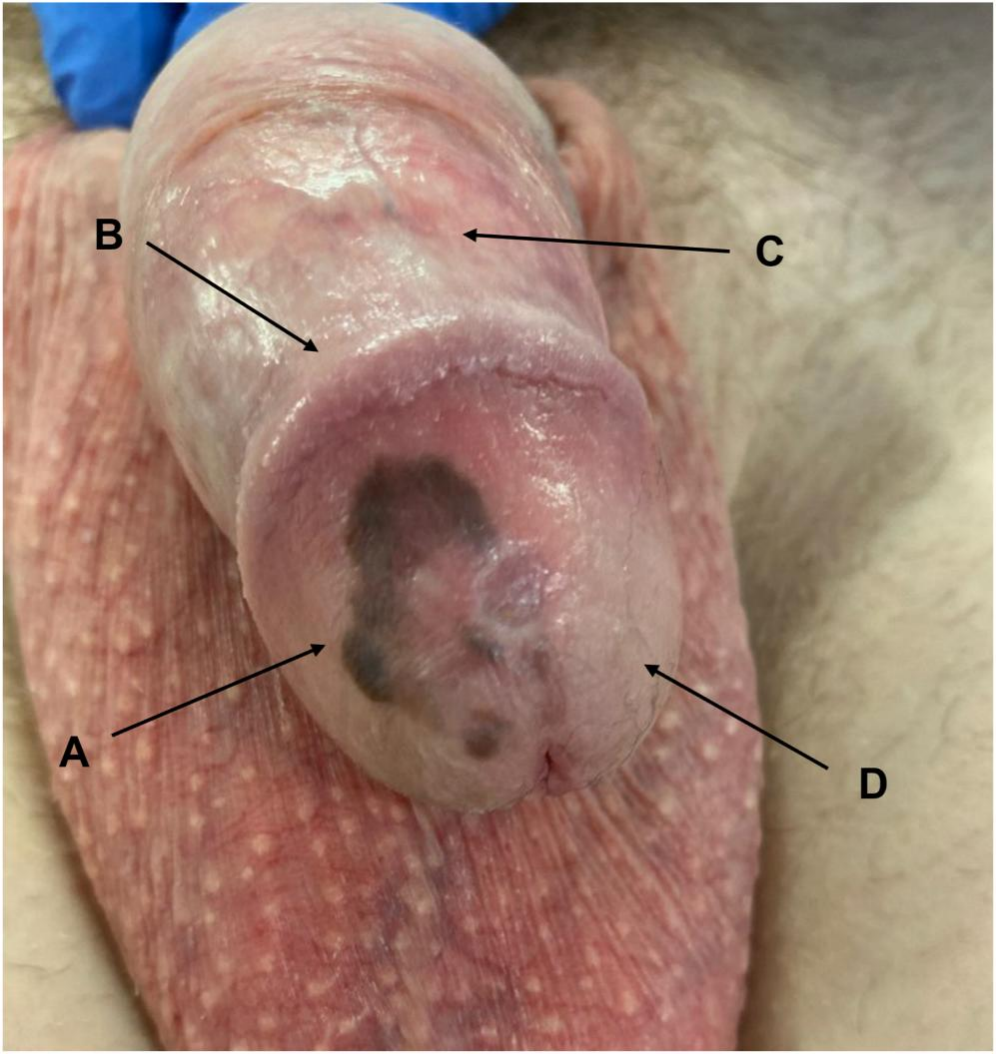
Clinical photograph of a 45‐year‐old man with superficial spreading melanoma of the penis and lichen sclerosus, diagnosed both clinically and histologically. (a) Melanoma; (b) Effacement of the coronal sulcus; (c) Sclerosis; (d) Etiolation of the glans.

Overall, by compounding the findings from histology, photos, and medical notes, a total of nine patients (82%) were found to have features of MGLSc (Figures 1, 2, 3).

## 4 DISCUSSION

PeMM is a rare entity accounting for 0.7% of penile cancer (PeCa) and 0.1% of MM cases.^6^ Similarly, vulval (Vu) MM accounts for only 1% of all MM in females.^7^ Like other malignancies, MM most probably represents a multifactorial disease due to a combination of environmental factors and altered host responses contributing to an alteration of regulatory processes. Several risk factors have been associated with cutaneous MM, including intermittent exposure to ultraviolet (UV) radiation, history of sunburn, the presence of atypical naevi, specific skin phenotypes and a family history of MM, and the use of psoralens and ultraviolet A therapy (PUVA) over a long period of time. Although UV exposure is important in the pathogenesis of cutaneous MM, the cause of genital disease is obscure and specific risk factors for genital MM have not yet been elucidated.^8^

MGLSc is an acquired, chronic, inflammatory skin disease associated with significant sexual dysfunction (dyspareunia), urethral involvement and penile (Pe) SCC.^1^, ^2^, ^9^ There are wide ranges in the estimates of the incidence of MGLSc, from 0.07% to 0.3%.^2^, ^10^, ^11^, ^12^ However the condition is likely to be under‐reported by patients as well as under‐recognised by clinicians.^2^, ^13^

It is now well‐recognised that MGLSc is associated with *squamous* malignancy. MGLSc is associated with penile intraepithelial neoplasia (PeIN), and this is a precursor of PeSCC. The estimated risk of *frank* PeSCC in patients with MGLSc ranges from 0% to 12.5%.^1^, ^9^, ^14^, ^15^, ^16^, ^17^, ^18^ The most likely pathogenesis is chronic inflammation and scarring due to MGLSc. This phenomenon is well‐documented at other sites and with other diseases.^19^ Potent topical steroid treatment does not seem to be an important risk in men and women (rather, the opposite), but there is limited data on the long‐term safety of topical calcineurin inhibitors^.^ ^9^, ^14^, ^20^, ^21^

To our knowledge, there have only been three previously documented cases (all from our group), of PeMM coexisting with GLSc.^5^ We remarked then that the PeMM that we observed complicating prior GLSc may have been coincidental in these cases, however we did note that in the 22 years of running a male genital dermatology service, these were the only cases of PeMM that had been encountered; in other words, no MM had been seen by us in the absence of MGLSc.^5^

In addition to being under‐recognised clinically, GLSc in the context of MM might also be under‐recognised histologically. In this study, we noticed that the features of LSc tended to vanish beneath the tumour itself, where dermal hyalinisation was replaced by fibrosis. This is an interesting phenomenon that has also been reported in the literature. While melanocytic naevi can be found coinciding with GLSc, the histologic changes of LSc are often only found at the periphery of the MM but not immediately adjacent to or underlying the tumour itself.^4^ This fact complicates the histological diagnosis of LSc and could account, at least partially, for the low numbers of cases on the topic.

We have very recently reviewed the literature on the association of MM with GLSc.^4^ In contrast to the literature on male genital MM, there have been several reports of VuMM occurring in females with Vu LSc, particularly in the paediatric population.^7^, ^22^, ^23^, ^24^, ^25^ A study in 2019 identified 249 patients with Vu LSc (confirmed on biopsy), of whom three also had VuMM. The same study reported that 30 cases of SCC were found among patients with LSc. Conversely, six cases of MMs were diagnosed among patients who did not have LSc from a total of 250,000 females. The overall risk of VuMM for female patients with LSc was thus estimated to be 341 times higher than the risk for women without LSc (*p* < 0.001).^26^

VuMM associated with LSc is uncommon but is more often reported in the paediatric population than the adult population.^7^, ^8^, ^27^, ^28^, ^29^, ^30^, ^31^ Our tertiary service is limited to those over the age of 16 years, and our results could be skewed regarding the overall age of patient with PeMM. The prevalence of paediatric PeMM in MGLSc is not known as the literature is devoid of cases on the topic.

This work adds to our previous case reports by further highlighting the possible association between MGLSc and PeMM.^5^ Our present work has found MGLSc in the majority (9/11; 82%) of all PeMM cases seen at our tertiary service over an 11‐year period. The pathogenesis of PeMM arising in MGLSc is not clear, nor is the temporal association between the two conditions, though it is presumed that the chronic, more subtle changes seen in MGLSc pre‐date the MM.

As UV radiation, the only proven environmental ‘melanocyte carcinogen’, cannot credibly be a causative factor in MM of the genital skin, it seems plausible that local stressors are responsible for the development of MM in these locations. Melanocytic dysfunction and distress are seen in and after chronic MGLSc, manifesting as penile melanosis, and there is a literature attesting to the challenges this can create in the histological differential diagnosis.^1^, ^27^, ^28^, ^29^, ^30^ It is not unreasonable to postulate that chronic melanocytic distress in MGLSc can lead to MM.

LSc may be responsible for generating a pro‐oxidative environment increasing the risk of mutations, whilst altering the extra‐cellular matrix composition leading to clonal expansion of damaged melanocytes.^31^ Finally, the dense subepidermal fibrosis‐sclerosis may prevent the function of T‐cytotoxic T lymphocytes allowing for expansion of melanocytes with an abnormal phenotype.^31^ It is not clear from our cases whether melanocytic lesions (*e*.*g*., naevi or penile melanosis) were present prior to the development of the malignant lesion. Due to what we already suspect about the under‐recognition and under‐reporting of MGLSc, it is likely that it may be far more prevalent than realized, and thus only detected when specifically sought out, as in this case series.

The exact molecular mechanisms that may be involved in this process are not clear. It is possible that events involved in transformation of MGLSc to PeSCC are also relevant in the development of melanoma within LSc. Increased Wnt/β‐catenin signalling is observed in PeCa, and *β*‐catenin has been shown to play a critical role in the early stages of melanocyte transformation; however, the exact role of WNT‐signalling in melanoma initiation and progression remains unclear.^32^, ^33^

HPV has been inculpated in the pathogenesis of VuMM, fascinatingly non‐genital HPV types.^34^ This requires further investigation in both sexes. Human endogenous retroviruses (HERV) have been implicated in the pathogenesis of MM and at cutaneous sites photoactivation has been mooted mechanistically.^35^, ^36^ Chronic inflammation or chronic exposure to ultrapotent topical steroids might speculatively be incriminated in genital HERV reactivation.

An alternative explanation for our findings, but one that we favour less, would be that the anatomical changes caused by a primary MM of the penis may predispose to the chronic inflammation leading to MGLSc. However, this seems unlikely given the MM would need to be present for some time prior to the LSc becoming apparent both histologically and clinically.

This study is limited by its size and retrospective nature of data collection. By nature of the study design, the histopathology findings may be distorted by subjectivity. We attempted to counter this by using two histopathologists to review the histology independently, with a third histopathologist recruited to address any disconcordant findings. Despite our best efforts to identify all cases of PeMM presenting to our tertiary service, there may have been cases that we have not identified, although this is unlikely since all multi‐disciplinary team meetings in the time frame to be included were searched and it is highly likely that all cases of PeMM would have been discussed.

## 5 CONCLUSION

The histopathological confirmation of the presence of LSc in nine out of eleven men with PeMM seen at our tertiary centre does not prove a causative relationship between GLSc and PeMM but is suggestive. We theorise that this is related to chronic melanocytic distress created by LSc. Our findings have implications for the prevention, early diagnosis, management, and follow‐up of MGLSc.

## AUTHOR CONTRIBUTIONS

**Kate Dear**: Data curation (lead); formal analysis (equal); methodology (equal); project administration (lead); writing—original draft (lead); writing—review and editing (equal). **Georgios Kravvas**: Conceptualization (equal); formal analysis (equal); methodology (equal); supervision (supporting); writing—review and editing (equal). **Sharmaine Sim**: Writing—review and editing (equal). **Evanthia Mastoraki**: Investigation (equal); writing—review and editing (equal). **Mariel James**: Writing—review and editing (equal). **Richard Watchorn**: Writing—review and editing (equal). **Aiman Haider**: Conceptualization (equal); data curation (equal); investigation (equal); methodology (equal); writing—review and editing (equal). **Peter Ellery**: data curation (equal); methodology (equal); writing—review and editing (equal). **Alex Freeman**: Data curation (equal); Writing—review and editing (equal). **Hussain M. Alnajjar**: Methodology (equal); writing—review and editing (equal). **Asif Muneer**: Writing—review and editing (equal). **Christopher B. Bunker**: Conceptualization (lead); formal analysis (equal); investigation (equal); methodology (equal); supervision (lead); writing—review and editing (lead).

## CONFLICT OF INTEREST STATEMENT

None to declare.

## ETHICS STATEMENT

Not applicable.

## Data Availability

Data available upon reasonable request.
